# The SARS-CoV-2 N Protein Is a Good Component in a Vaccine

**DOI:** 10.1128/JVI.01279-20

**Published:** 2020-08-31

**Authors:** Gustaf Ahlén, Lars Frelin, Negin Nikouyan, Friedemann Weber, Urban Höglund, Olivia Larsson, Marie Westman, Ola Tuvesson, Eva-karin Gidlund, Matteo Cadossi, Sofia Appelberg, Ali Mirazimi, Matti Sällberg

**Affiliations:** a Department of Laboratory Medicine, Division of Clinical Microbiology, Karolinska Institutet, Stockholm, Sweden; b Justus Liebig University Giessen, Giessen, Germany; c Adlego Biomedical AB, Uppsala, Sweden; d Karolinska Trial Alliance, Karolinska University Hospital, Stockholm, Sweden; e Cobra Biologics, Matfors, Sweden; f IGEA Spa, Carpi, Italy; g Public Health Agency of Sweden, Solna, Sweden; University of Kentucky College of Medicine

**Keywords:** COVID-19, DNA vaccine, SARS-CoV-2, vaccine

## LETTER

**FIG 1:**
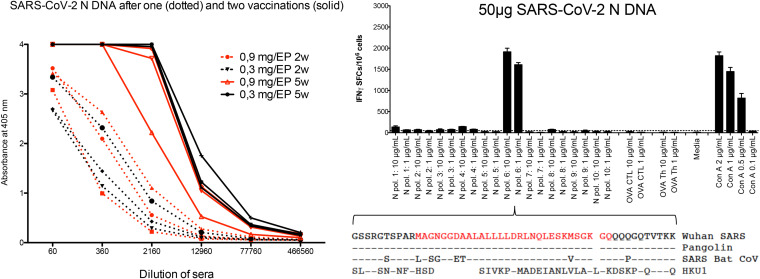
Immunization of groups of three rabbits with 0.3 or 0.9 mg of SARS-CoV-2 N DNA (i.m.) using *in vivo* EP at weeks 0 and 3, with venous blood obtained at weeks 2 and 5. Results from testing antibody levels to N by ELISA is shown to the left. Also shown (upper right) is the immunization of groups of 5 C57BL/6 mice with 50 μg SARS-CoV-2 N DNA (i.m.) using *in vivo* EP and analysis of T-cell responses by an IFN-γ ELISpot. Data are given as the number of spot-forming cells/million splenocytes. Also shown is the sequence of the four overlapping peptides in the reactive peptide pool and the alignment with the indicated coronaviral sequences (lower right). The amino acids in red indicates the likely epitope region.

Dutta and coworkers suggest in a recent letter (1) that the severe acute respiratory syndrome coronavirus 2 (SARS-CoV-2) nucleoprotein (N) might be a good vaccine target. They argue that the conserved nature of the N protein makes it a suitable vaccine component. The concept of using a nucleoprotein to protect against infection was already shown in chimpanzees in 1985 when Iwarson and colleagues used the hepatitis B core antigen to protect chimpanzees against hepatitis B challenge (2). A SARS-CoV-2 infection in macaques protects against reinfection, supporting the concept of a protective immunity (3), and an inactivated whole-virus vaccine, containing all structural proteins of SARS-CoV-2, protects macaques against infection (4). However, the vast majority of vaccines currently in clinical development are based only on the spike protein, or parts thereof, and seem to protect against disease but not against infection (5, 6). When these are based on viral vectors, antivector immunity limits repeat vaccinations. We agree with Dutta and colleagues on the importance of the N protein in vaccines and show data to support this view. The partners in the OPENCORONA vaccine consortium generated a codon-optimized SARS-CoV-2 N gene based on the Wuhan-1 isolate (7) (GenScript, USA). A final SARS-CoV-2 vaccine combines the N protein with other structural proteins to generate a synthetic whole-virus vaccine. To first test that a SARS-CoV-2 N plasmid is safe and immunogenic in a larger animal, we immunized six New Zealand White rabbits with 0.3 or 0.9 mg of DNA intramuscularly (i.m.) at weeks 0 and 3 using *in vivo* electroporation (EP) (GeneDrive; IGEA, Italy). Venous blood was drawn at weeks 2 and 5 and was analyzed for the presence of N antibodies by an in-house enzyme-linked immunosorbent assay (ELISA) (8) using an Escherichia coli-expressed N protein based on the same strain (GenScript). A single injection of the N plasmid induced anti-N titers of 10^3^ to 10^4^, and 2 weeks after a boost the levels reached 10^4^ to 10^5^, with no difference in the DNA dose used (Fig. 1). Thus, the SARS-CoV-2 N gene was safe and highly immunogenic as a DNA vaccine. To evaluate the ability of the SARS-CoV-2 N DNA to induce T cells, and in particular T cells cross-reacting with coronaviruses (CoVs) from other species, we immunized groups of C57BL/6 mice with N protein in adjuvant (data not shown) or 50 μg of DNA. Splenocytes were analyzed for recognition of N-based peptide pools containing four overlapping peptides by enzyme-linked immunospot (ELISpot) assay as described previously (8). This revealed a single region to which H-2^b^-restricted T cells produced both interleukin-2 (IL-2) (data not shown) and gamma interferon (IFN-γ) (Fig. 1). Importantly, the sequence of this region was 100% identical to that of pangolin CoV and had an 86% homology with that of bat CoV. Thus, although this is a murine T-cell epitope, this predicts the benefit of using SARS-CoV-2 vaccines that also include the N protein, as this protein is likely to induce immune cells that also recognize CoVs in future outbreaks. Hence, including N and/or other structural proteins in addition to spike-related sequences adds the benefits of increasing immunogenicity and ensures a more future-proof vaccine design.
